# A Dual Role for Abscisic Acid Integrating the Cold Stress Response at the Whole-Plant Level in *Iris pseudacorus* L. Growing in a Natural Wetland

**DOI:** 10.3389/fpls.2021.722525

**Published:** 2021-12-07

**Authors:** Vicent Caselles, Andrea Casadesús, Sergi Munné-Bosch

**Affiliations:** ^1^Department of Evolutionary Biology, Ecology and Environmental Sciences, Faculty of Biology, University of Barcelona, Barcelona, Spain; ^2^Research Biodiversity Institute, Faculty of Biology, University of Barcelona, Barcelona, Spain

**Keywords:** ABA, cold stress, *Iris pseudacorus* L., leaf senescence, perennial, rhizome, winter

## Abstract

Leaf senescence, the last stage of the developmental program of leaves, can be induced by both internal and external signals. Cold stress-induced leaf senescence is an efficient strategy to overcome winter temperatures. In this work, we studied leaf senescence in yellow flag (*Iris pseudacorus* L.) individuals growing in a natural wetland, not only considering its relationship with external and internal cues, but also the plant developmental program, and the biological significance of rhizomes, storage organs that remain viable through winter. Total chlorophyll contents and the maximum efficiency of PSII (*F_v_*/*F_m_* ratio) decreased in senescing leaves, which was associated with a sharp increase in abscisic acid (ABA) contents. Furthermore, total cytokinin and 2-isopentenyladenine contents decreased in December compared to November, as plants became more stressed due to a decline in air temperatures. ABA increases in senescing leaves increased in parallel to reductions in violaxanthin. Rhizomes also accumulated large amounts of ABA during winter, while roots did not, and neither roots nor rhizomes accumulated 9-*cis*-epoxycarotenoids, thus suggesting ABA, which might play a role in conferring cold tolerance to this subterranean organ, may result from phloem transport from senescing leaves. It is concluded that (i) leaf senescence is a highly regulated physiological process in yellow flag playing a key role in the modulation of the entire plant developmental program, and (ii) ABA plays a major role not only in the regulation of leaf senescence but also in the establishment of cold tolerance in rhizomes, two processes that appear to be intimately interconnected.

## Introduction

Plants, being sessile organisms in their post-embryonic development, have developed several strategies to cope with environmental stresses. Among them, leaf senescence, which allows a progressive dismantling of the photosynthetic apparatus and a recycling of its components, includes not only photoassimilates but also amino acids, amides, glutathione, and other nutrient-rich compounds, thus helping promote survival at the organism level (Munné-Bosch and Alegre, 2004; Fischer, 2012). This process is a key phase in a plant’s development program as it represents the last stage of leaf development and is therefore tightly regulated by internal and external cues (Gan and Amasino, 1997).

Cold stress is characterized by an alteration of the physicochemical properties of key cellular components such as membrane lipids and enzymes, leading to changes in membrane fluidity and eventual damage to membranes, solute leakage, and a dysregulation of metabolic reactions due to alterations to enzymatic properties, which in turn lead to the production of reactive oxygen species (Welti et al., 2002). Being the last stage of leaf development, cold-induced leaf senescence is tightly regulated at several levels and can serve a role in acclimation (Masclaux-Daubresse et al., 2007). Indeed, depending on the influence of other environmental factors, such as photoperiod, light intensity, and quality, the impact of low temperature stress on plants largely varies (Novák et al., 2021). Therefore, carrying out experiments in plants growing in their natural habitat, although complex due to changing environmental conditions, is essential to understand better the mechanisms underlying cold stress tolerance in nature.

Plant hormones act as signaling molecules and exert the function of stress response and senescence regulation. ABA has been reported to act as the main phytohormone involved in stress signaling, since it induces stomatal closure and osmolyte accumulation in plants under environmental stresses such as drought and chilling stress (Daszkowska-Golec and Szarejko, 2013). Furthermore, it plays a pivotal role in leaf senescence induction and it has been reported that ABA contents increase during this process in several species (Gepstein and Thimann, 1980). ABA is synthesized from epoxycarotenoids such as neoxanthin and violaxanthin (Gepstein and Thimann, 1980), which are metabolized to obtain xanthoxin by the 9-*cis*-epoxycarotenoid dioxygenase (NCED) enzyme (Schwartz et al., 1997). Xanthophylls, besides being the precursors of ABA, are involved in the protection of the photosynthetic apparatus carrying out the process known as the xanthophyll cycle, which helps dissipate excess energy in the chloroplast through thermal dissipation (Thiele and Krause, 1994). On the other hand, cytokinins (CKs) play an antagonistic role to ABA in regulating leaf senescence. They have been reported to inhibit chloroplast degradation in senescent leaves, as they promote plant vigor and cellular division therefore delaying leaf senescence (Joshi et al., 2019). Furthermore, cytokinins regulate nutrient recycling during leaf senescence by participating in the establishment of source–sink relations; the contents of this phytohormone decrease as senescence progresses in leaves, while they are present at high concentrations in sink tissues during nutrient mobilization from sources (senescing leaves) to sinks (roots, storage organs, or reproductive tissues; Roitsch and Ehneß, 2000).

*Iris pseudacorus* L. is an angiosperm that grows in habitats with high soil water content. Its roots are usually submerged in water, and leaves emerge from the surface. Due to its low prevalence in high-altitude regions, *I. pseudacorus* is believed to be sensitive to low temperatures (Sutherland, 1990). When temperatures start to decline, *I. pseudacorus* starts a process of leaf senescence which culminates in December–January. At the beginning of spring, leaves regrow from the rhizome, which is a very desiccation-tolerant tissue (Washington State Noxious Weed Control Board, 2013). Given that, to the best of our knowledge, the literature describing leaf senescence processes at a whole-plant level in wetland plants is very scarce, we aimed to elucidate how *I. pseudacorus* regulates the senescence process that its leaves undergo during the last months of the year, while describing the intertwined physiological response of non-photosynthetic underground tissues. Particular attention was put on rhizomes, which specifically are those organs staying viable through winter and, when spring arrives, start the process of leaf regrowth, thus allowing perenniality and most importantly plant survival during winter.

## Materials and Methods

### Study Species and Experimental Design

*Iris pseudacorus* L., or yellow flag, is an angiosperm with stiff and erect leaves that emerge from the water. It mainly inhabits water communities, and it can be found both with its leaves partially submerged in the water or growing in the banks of water masses. It is considered as a plant with great invasive potential, since it spreads with ease both through its seeds and *via* vegetative propagation through its rhizomes, which can range from 1cm to 4cm in diameter and form thickets that hinder the growth of other species’ seedlings (Sutherland and Walton, 1990). *Iris pseudacorus* leaves undergo a senescence process that culminates during the winter and, later, in the early spring, new leaves re-grow from the rhizome, which remains as a perennial subterranean organ. Flowering occurs in May–June, resulting in bright yellow flowers.

**Figure 1:**
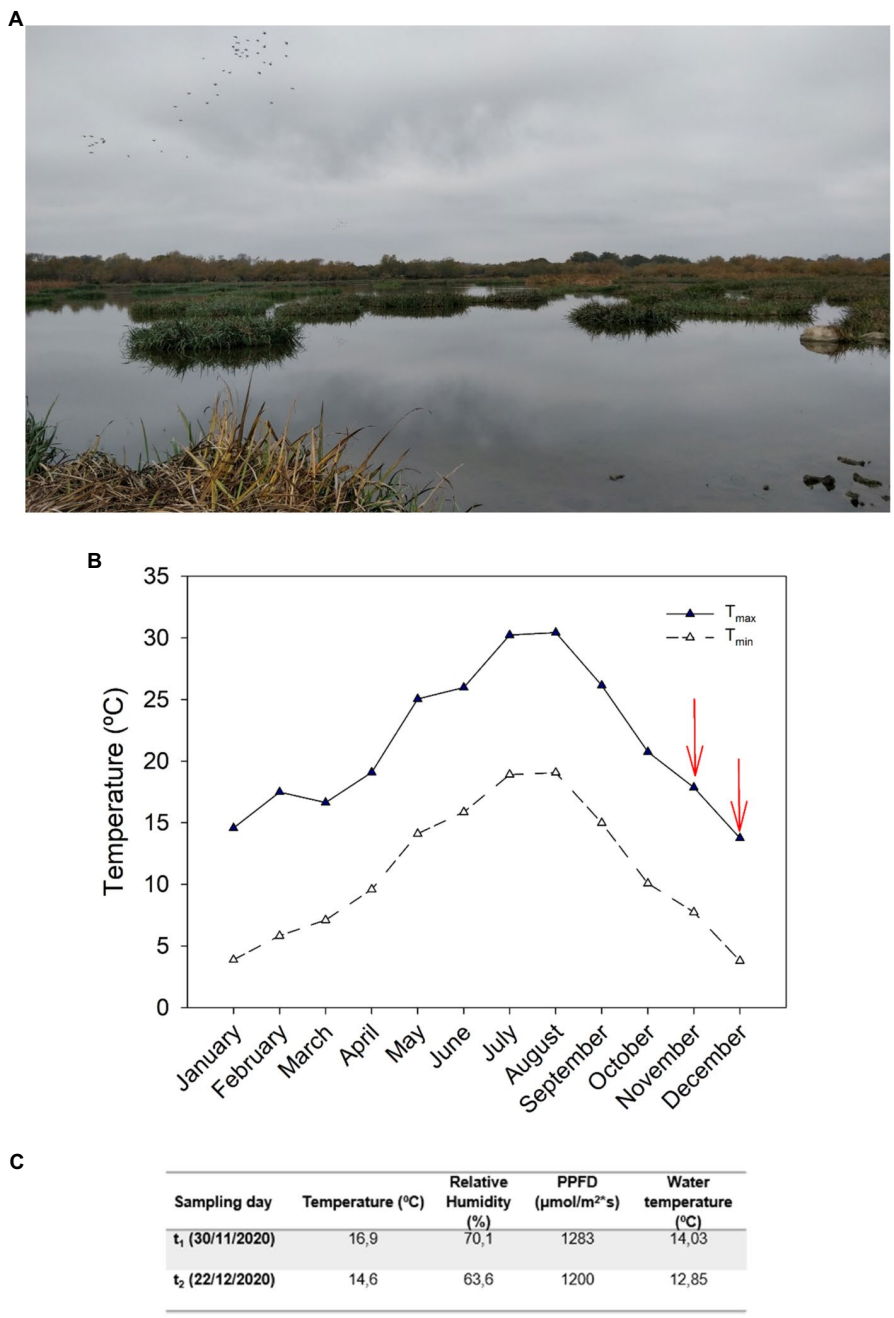
Ecosystem description and climatological data from the study site. **(A)** Image taken from the study site at *Estany de Vilaüt* (42.283 N, 3.117E) located in the northeast of Catalonia, Spain. *Iris pseudacorus* mats can be seen in the water. **(B)** Maximum and minimum average monthly temperatures during 2020 recorded by the *Servei Meteorològic de Catalunya* meteorological station located closest to the study site, in *Castelló d’Empúries* (42.260 N, 3.074E). **(C)** Temperature, relative humidity (RH), photosynthetic photon flux density (PPFD), and water temperature at midday during the two sampling days (November 30 and December 22, 2020) recorded at the site of study.

An experiment with *I. pseudacorus* L. growing under natural conditions in the wetlands located in the lake *Estany de Vilaüt* (42.283N, 3.117E; Figure 1A), in the province of Girona, Catalonia, Spain, was conducted during later autumn and the beginning of winter 2020, a period of the year characterized by marked phenological changes associated with cold-induced leaf senescence. This location is characterized by mild winters, with minimum registered temperatures of 0 and −1°C in November and December (and minimum average monthly temperatures were of 7 and 3°C in November and December, respectively, Figure 1B). Climatic data for 2020 were provided by the *Servei Meteorològic de Catalunya*, recorded at the closest meteorological station to the study site, located near *Castelló d’Empúries* (42.260 N, 3.074E). To study the processes regulating leaf senescence in response to cold stress at a whole-plant level, two samplings were conducted during November and December 2020. The first sampling was conducted on November 30, 2020, and the temperature recorded at the time of sampling was 16.9°C, with a photosynthetic photon flux density (PPFD) of 1,283 μmol/m^2^s, a relative humidity (RH) of 70.1%, and water temperature of 14.3°C. The second sampling was conducted on December 22, 2020, and at the time of sampling the temperature was 14.6°C, with a PPFD of 1,200 μmol/m^2^ s, a RH of 63.6, and 12.9°C water temperature (Figure 1C). Both samplings were conducted at solar midday to ensure comparable responses.

**Figure 2:**
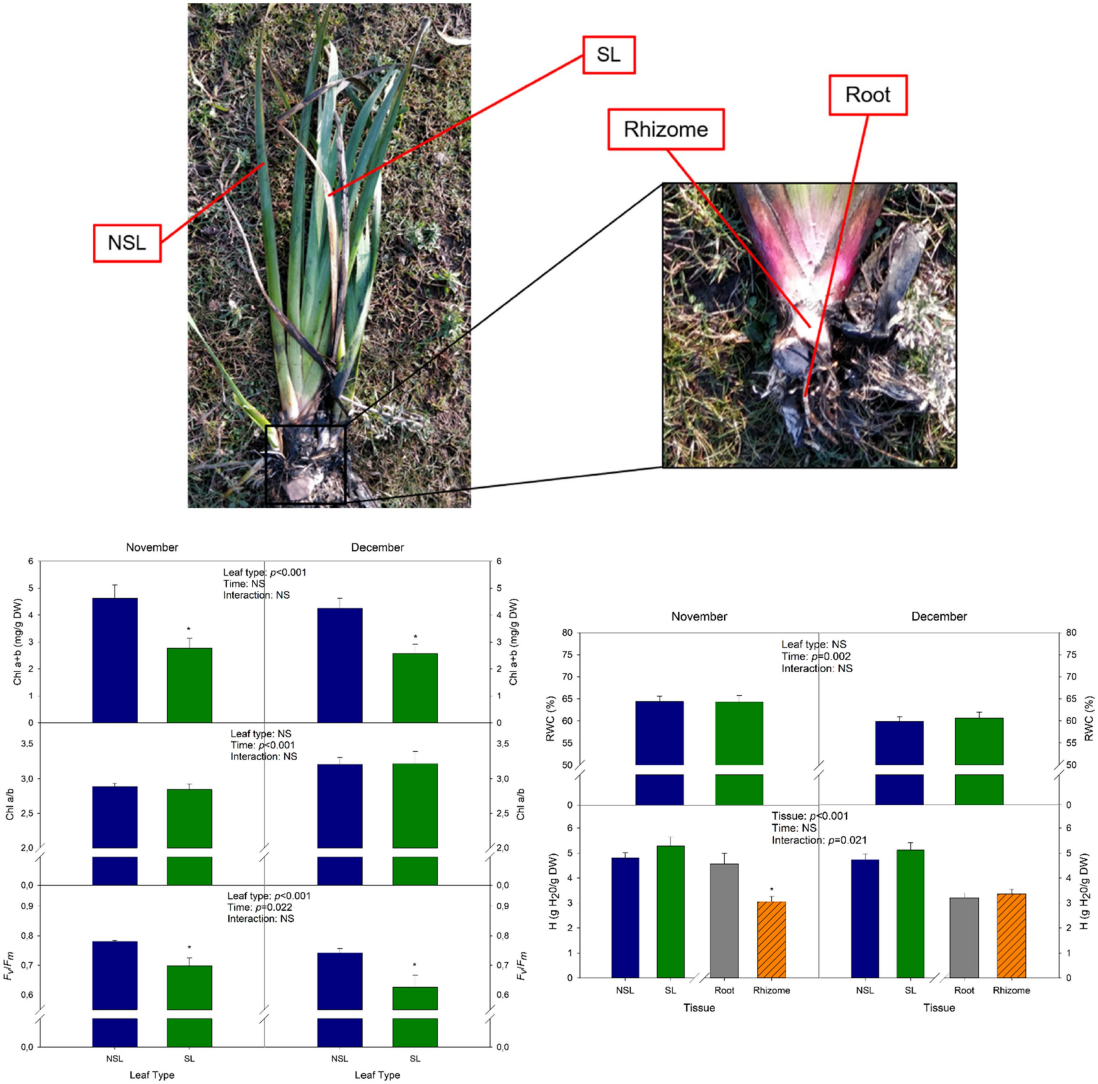
Cold stress-induced leaf senescence in *Iris pseudacorus* individuals at the start of winter. Phenotype of senescent leaves (SLs) and non-senescent leaves (NSLs) sampled in this study, with a close-up of a rhizome and the root system of *I. pseudacorus*. Total chlorophyll contents (Chl a+b), chlorophyll a-to-b ratio (Chl a/b), and maximum efficiency of PSII (*F_v_/F_m_*) of SL and NSL leaves sampled in November and December. The relative water content (RWC) of SLs and NSLs, and the hydration (H) of SLs, NSLs, roots, and rhizomes are also shown. Values of *p* from the two-way mixed factorial analysis are shown in the inlets, including the factor *Time*, *Leaf type/Tissue*, and their interaction. An asterisk indicates differences between leaf types or root tissue types. Data show the mean±SE of *n*=12 individuals. NSL, non-senescent leaf; SL, senescent leaf.

In each sampling, 12 homogenous *I. pseudacorus* L. mature individuals were randomly selected and sampled. Two leaves were selected for each individual based on their phenotype: a senescent leaf (SL) and a non-senescent one (NSL; Figure 2). For each leaf type, a sample was immediately frozen in liquid nitrogen and then stored at −80°C until the biochemical analysis, and another one was used to assess physiological parameters such as *F_v_*/*F_m_* and relative leaf water content (RWC). Additionally, samples from subterranean organs were collected, including both the rhizome, henceforth called “rhizome,” and the root system, from now on called “root” (see Figure 2). A sample from each organ was frozen in liquid nitrogen as described before, and another one was used for the determination of physiological parameters such as the water content.

The physiological parameters assessed for each leaf were RWC, hydration (H), and maximum efficiency of PSII (*F_v_/F_m_*); and for the non-photosynthetically active organs (rhizome and root), hydration (H) was determined. The biochemical parameters analyzed were the chlorophyll, carotenoid, and hormone contents.

### Water Contents and *F*_v_/*F*_m_ Ratio

Relative water content was determined as (FW−DW)/(TW−DW)×100, where FW means fresh weight, TW means turgid weight and was measured after 24 h submerged in water at 4°C, and DW means dry weight and was obtained by oven-drying at 70°C until constant weight. Hydration was calculated as (FW−DW)/DW. The maximum efficiency of PSII (*F_v_/F_m_*) was measured in dark-adapted leaves using a portable chlorophyll fluorimeter (Mini-PAM II Photosynthesis Yield Analyser, Walz, Germany).

### Chlorophyll Contents

Fifty micrograms of leaf sample were ground in liquid nitrogen and extracted with 0.5 ml of cold methanol with 0.01% butylated hydroxytoluene (BHT), using ultrasonication for 30min (Bransonic ultrasonic bath 2800, Emerson Industrial, Danbury, CT, United States) and vortexing before and after ultrasonication. Afterward, extracts were centrifuged for 10min at 13,000rpm and 4°C (centrifuge MR18-22, Jouan, Saint-Herblain, France), and the supernatant was collected. The pellet was re-extracted twice with 0.5 ml of methanol as described before, and the supernatants were pooled, equating to a final extract volume of 1.5 ml. Then, samples were filtered using 0.22- μm PTFE filters (Phenomenex, Torrance, CA, United States). Before spectrophotometric analysis, samples were diluted 1:10 (v/v) with pure methanol and absorbances were then read at 470, 653, 666, and 750 nm using an UV/visible spectrophotometer (Shimadzu UV-160A, Shimadzu, Kyoto, Japan), and chlorophyll contents were calculated using the equations described in Lichtenthaler and Wellburn (1983).

### Carotenoid Contents Determination

The quantification of carotenoids was carried out by HPLC as described in Munné-Bosch and Alegre (2000). Briefly, the extraction was carried out as described in the previous section, using methanol with 0.01 BHT as solvent. Samples were injected into reverse-phase HPLC, separated using a binary-solvent gradient (A: acetonitrile/methanol, 85:15, v/v; B: methanol/ethyl acetate, 68:32, v/v) and quantified with a diode array detector at 445 nm. The de-epoxidation state (DPS) of the xanthophyll cycle was calculated as follows: DPS=(Zx+0.5Ax)/(Vx+Ax+Zx) (Thayer and Björkman, 1990).

### Determination of Phytohormones

Phytohormone contents (ABA, *t*-Z, IPA, 2-IP, ZR, GA_1_, GA_3_, GA_4_, and GA_7_) were determined by UPLC-MS/MS as described in Müller and Munné-Bosch (2011). The extraction was carried out as described in the previous section, with the addition of deuterium-labeled internal standards at the beginning of the process. Quantification was carried out using the recovery rate of the labeled hormones and by the construction of calibration curves for each analyte. To do so, the software Analyst (Applied Biosystems, Inc., California, United States) was used.

### Statistical Analyses

A two-way mixed factorial analysis was conducted to assess the effects “*Time*” and “*Leaf type*” or “*Tissue*” depending on the comparison being performed. To assess the differences between the two types of leaves (non-senescent vs. senescent leaf) and between the two types of subterranean organs (root, rhizome), Tukey’s *post-hoc* tests were carried out (*agricolae* package). Data were tested for homoscedasticity of variances using the Bartlett test and for normality using the Shapiro–Wilk test. For the data not in compliance with said conditions, the non-parametrical factorial analysis method *ART* (*ARTool* package) was used (Wobbrock et al., 2011). All data are represented as mean±SE. Differences are considered statistically significant if *p*<0.05. All statistical analyses were performed using the R statistical software (R Foundation for Statistical Computing, Vienna, Austria).

## Results

### Low Temperature Induces Leaf Senescence in *Iris pseudacorus*

To describe the process of senescence that leaves of *I. pseudacorus* growing under natural conditions undergo in response to low temperatures, two leaves were selected per individual during November: one with a non-senescent phenotype, which was used as a control, and one with a senescent phenotype (Figure 2). The *F_v_/F_m_* showed a decrease between the non-senescent leaf (NSL, control) and the senescent leaf (SL) in November, with a mean value under 0.75 for the latter (0.699; Figure 2B). *F_v_/F_m_* further decreased to 0.633 for the SL in December, consistent with the decrease in temperatures experienced with the onset of winter, as both the maximum and minimum monthly temperatures registered by the closest meteorological station (Figure 1B) and the air and water temperatures measured on site declined in December compared to November (Figure 1C). Total chlorophyll contents (Chl a+b) showed a similar pattern. In November, Chl a+b contents in NSL were 45% higher than those of SL. In December, the same pattern occurred (40% decrease between leaf types), but there were not any significant differences due to *Time* (Figure 2B). The senescence phenotype did not affect the chlorophyll a-to-b ratio (Chl a/b), as values were very similar between NSL and SL (2.89 and 2.84, respectively). However, in December Chl a/b increased, suggesting that, together with the results from *F_v_/F_m_*, individuals were suffering more stress and leaves showed a more advanced senescent stage in December compared to November (Figure 2B).

Regarding the water status of *I. pseudacorus* leaves, there were no differences between NSL and SL neither in November nor in December. However, a significant decrease due to the *Time* factor was observed, as RWC decreased by 7% in the NSL group and by 6% in SLs in December compared to November. There were no significant differences in H between leaf types neither in November (*p*=0.257 for the post-hoc analysis) nor in December (*p*=0.327). However, it is noteworthy that, in November, rhizomes showed significantly lower H than the root, yet in December both organs showed similar H (Figure 2C). Visual observations revealed that there was no re-greening of senescing leaves after winter stress and all aboveground organs in the next season were new sprouts.

### ABA Contents Increase in Senescing Leaves and in Rhizomes

**Figure 3:**
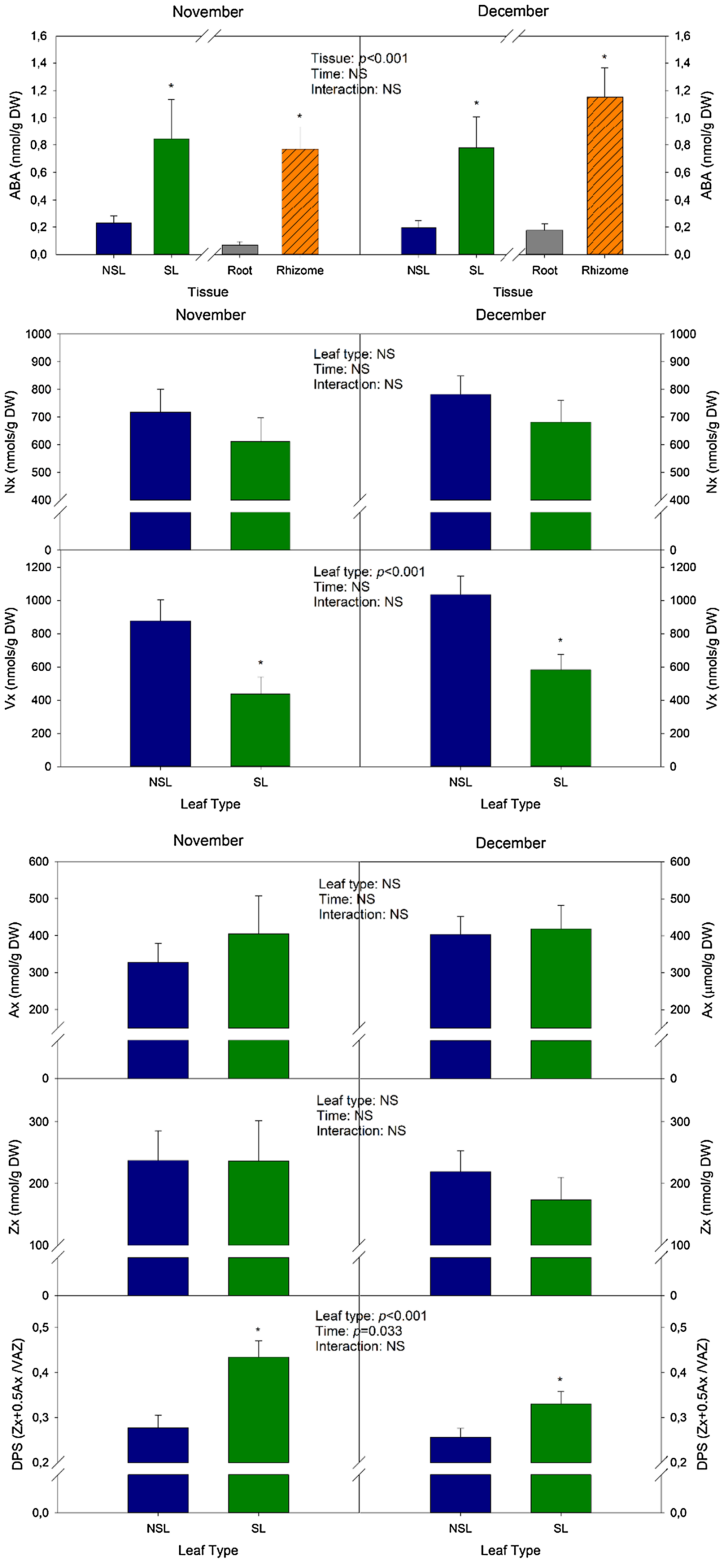
Changes in abscisic acid (ABA) and carotenoid contents during cold-induced leaf senescence. ABA, violaxanthin (Vx), neoxanthin (Nx), antheraxanthin (Ax), zeaxanthin (Zx) contents, and the de-epoxidation state of the xanthophyll cycle (DPS) of senescent and non-senescent leaves. Values of *p* from the two-way mixed factorial analysis are shown, including the factor *Time*, *Leaf type/Tissue*, and their interaction. An asterisk indicates differences between leaf types or root tissue types. Data show the mean±SE of *n*=12 individuals. NSL, non-senescent leaf; SL, senescent leaf.

Abscisic acid levels in the senescent leaf group were 73 and 75% higher than in the non-senescent one in November and December, respectively. The highest ABA contents recorded in this study belong to the SL from individuals sampled in November, with 3.6 nmol/g DW (Figure 3). As it can be seen in Figure 3, neoxanthin contents (Nx), an ABA precursor, did not vary significantly either between leaf types or times. However, values for the SL group tended to be lower than those of NSLs in average. Contrastingly, violaxanthin contents (Vx), another precursor of ABA biosynthesis, decreased in SL compared to NSL, by 50% in November and by 44% in December (Figure 3). The de-epoxidation state of the xanthophyll cycle (DPS), a parameter that quantifies the conversion of violaxanthin (Vx) to antheraxanthin (Ax) and zeaxanthin (Zx), was significantly higher in SL compared to NSL. Furthermore, there was a significant decrease due to *Time*, which is consistent with a more advanced senescent stage (Figure 3). Most notably, Ax and Zx contents, the de-epoxidated forms of the xanthophyll cycle, did not vary between both leaf types, thus indicating that the increase in DPS was caused by a decrease in Vx rather than by an increase of either Ax or Zx.

Rhizomes showed significantly higher ABA content compared to roots (Figure 3). This organ showed the highest mean values recorded in the experiment, with 1.2 nmol/g DW in December. HPLC quantification of carotenoids in roots and rhizomes showed that contents for these compounds were not detectable (data not shown), thus indicating that ABA accumulation in these tissues may not occur due to local synthesis from xanthophylls.

### Cytokinin Contents Decrease in Response to Reduced Temperatures in Winter

**Figure 4:**
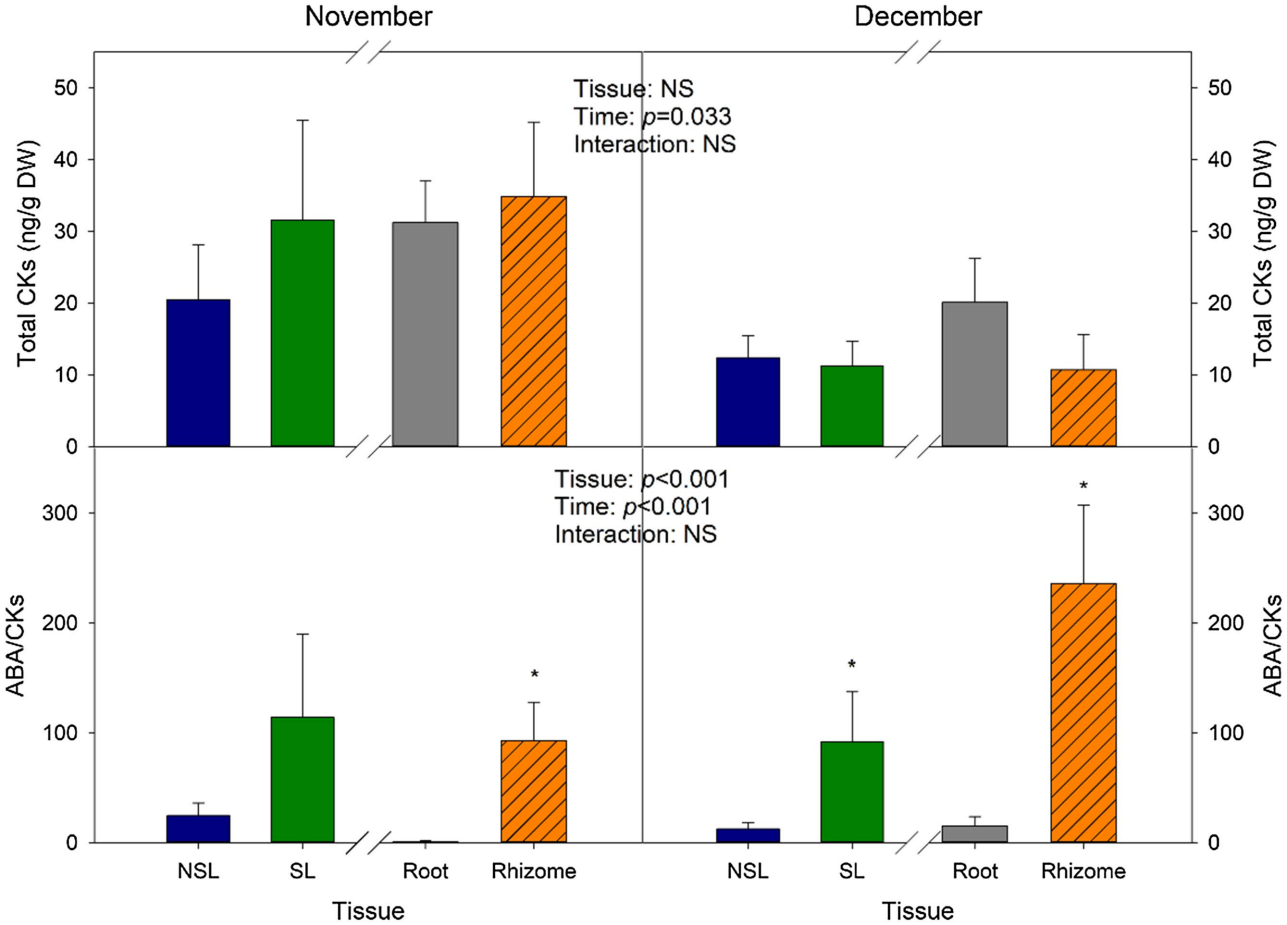
Cytokinin variations with time and between leaf type and belowground organ type. Total cytokinin levels (Total CKs) and ABA/CKs ratio in leaves (SL, NSL) and subterranean organs (roots and rhizomes) from *I. pseudacorus* individuals growing in natural conditions. Values of *p* from the two-way mixed factorial analysis are shown, including the factor *Time*, *Leaf type/Tissue*, and their interaction. An asterisk indicates differences between leaf types or root tissue types. Data show the mean±SE of *n*=12 individuals. NSL, non-senescent leaf; SL, senescent leaf.

To describe the last stage of the developmental program of *I. pseudacorus* leaves, we carried out a UPLC-MS/MS quantification of the contents of CKs, which have been reported to act antagonistically to ABA in regulating senescence. Total CK levels did not show variations due to the *Tissue* factor, as means for the leaves were 20.5 ng/g DW for the SL group and 31.6 ng/g DW for the NSL group, and for the subterranean organs, 34.9 ng/g DW and 31.2 for the root and rhizome, respectively (Figure 4). Nonetheless, levels decreased significantly in December compared to November (by 2-fold in NSL, SL, and rhizomes; and by 1.5-fold in roots), as individuals were suffering from greater stress given that temperatures declined at the start of winter. The ABA-to-CKs ratio (ABA/CKs) reflected these changes, as the ratio increased in December for the subterranean organs, reaching its maximum in rhizomes (average of 236.2). As could be expected, SL levels were higher than in NSL, despite these changes not being significant in the data from November. ABA/CKs in rhizomes were also significantly higher compared to roots (Figure 4).

**Figure 5:**
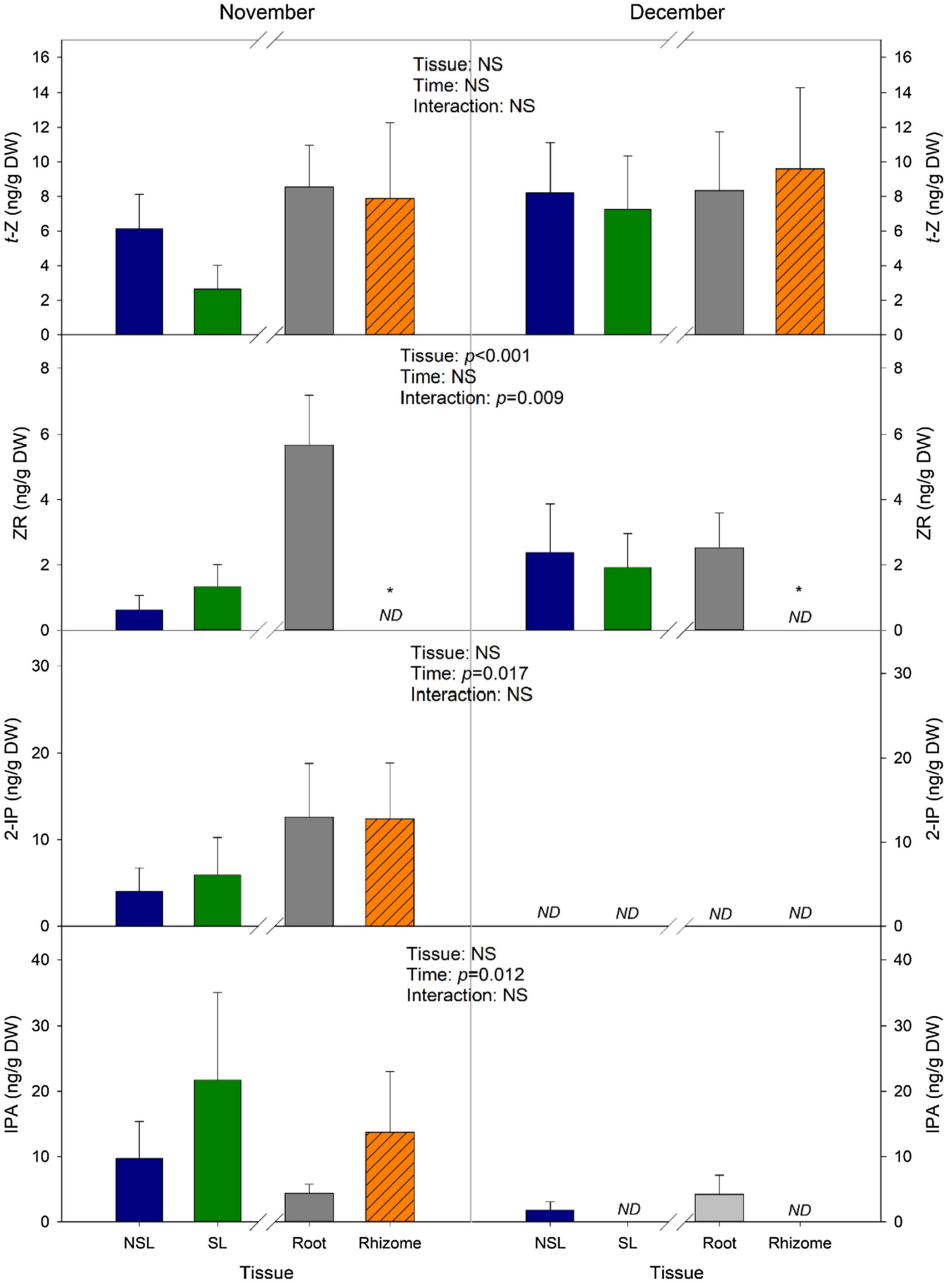
Changes in the concentration of individual cytokinins. Measurements were performed in leaves and subterranean organs of *I. pseudacorus*. Values of *p* from the two-way mixed factorial analysis are shown, including the factor *Time*, *Leaf type/Tissue*, and their interaction. An asterisk indicates differences between leaf types or root tissue types. Data show the mean±SE of *n*=12 individuals. NSL, non-senescent leaf; SL, senescent leaf; 2-iP, isopentenyladenine; iPA, isopentenyladenosine; *t*-Z, *trans*-zeatin; and ZR, zeatin riboside.

*trans-*Zeatin (*t-*Z) and 2-isopentenyladenine (2-iP) are the two main active forms of CKs, and their contents are displayed in Figure 5. A decrease in *t*-Z can be observed between leaf types in November; however, it is not statistically significant (*p*=0.134). Besides that, the average contents for this hormone did not show many variations in *Time* or *Tissue*, as values ranged between 6 and 9 ng/g DW (Figure 5). However, 2-iP contents, although they ranged between 4 and 5 ng/g DW on leaves and 12 ng/g DW in subterranean organs in the samples from November, were not detectable in neither leaves nor belowground organs from December (Figure 5), suggesting that the decrease in temperature in this month had an effect on the content of this active CK. As for the non-active CKs, zeatin riboside (ZR) levels in roots were higher than the levels in rhizomes in both months, as rhizomes showed non-detectable contents. Contents in leaves remained low, with mean values below 3 ng/g DW (Figure 5). Isopentenyladenosine (iPA) showed differences caused by *Time*, since values from December were below 4.3 ng/g DW (Figure 5).

### The ABA/GAs Ratio Increases in Rhizomes of Cold-Stressed Plants

**Figure 6:**
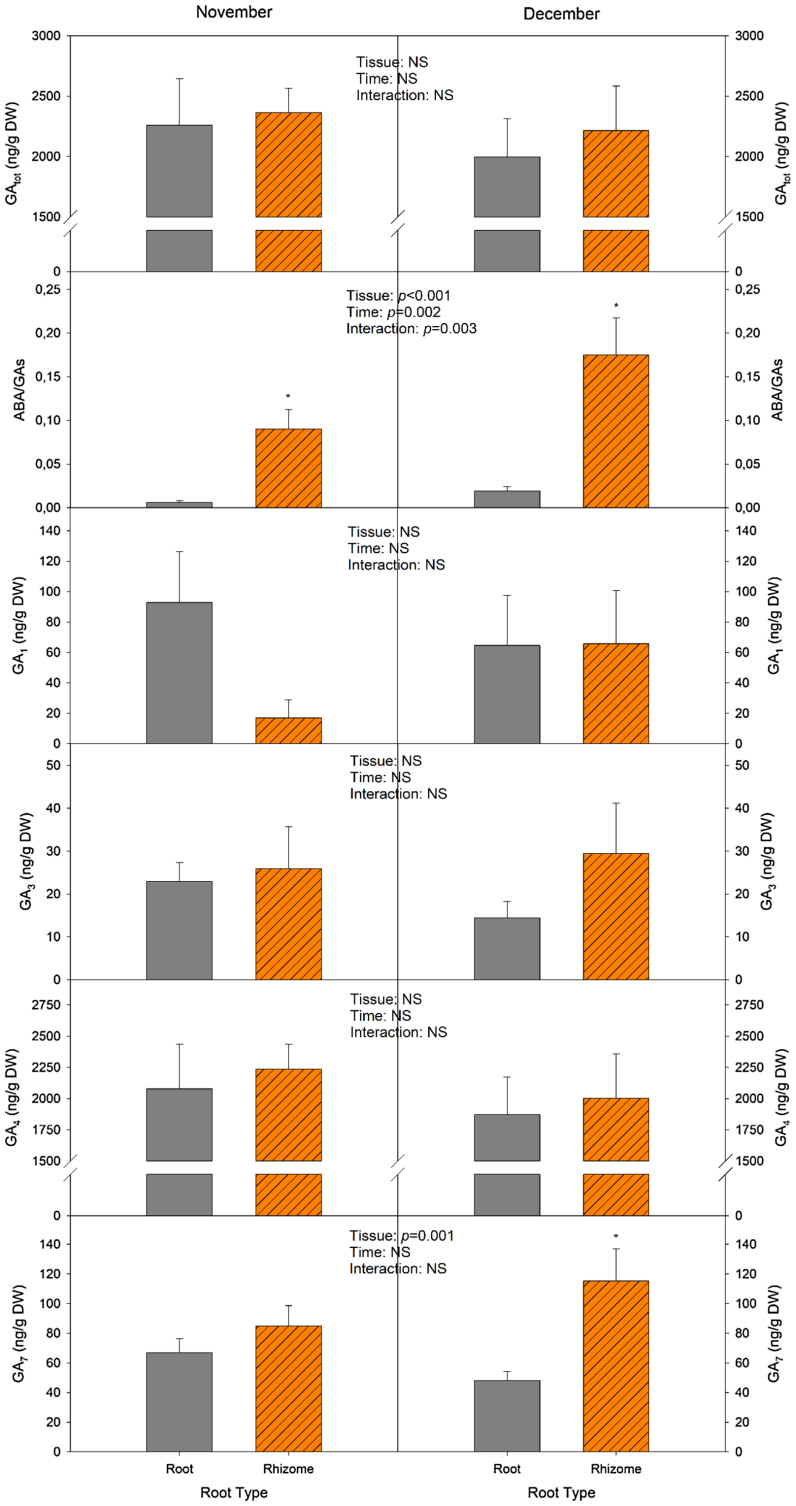
Changes in total gibberellin (GA_tot_) contents, ABA-to-GA ratio (ABA/GAs), and GA_1_, GA_3_, GA_4_, and GA_7_ concentrations. Measurements were performed in leaves and subterranean organs of *I. pseudacorus*. Values of *p* from the two-way mixed factorial analysis are shown, including the factor *Time*, *Leaf type/Tissue*, and their interaction. An asterisk indicates differences between leaf types or root tissue types. Data show the mean±SE of *n*=12 individuals. NSL, non-senescent leaf; SL, senescent leaf.

The most abundant GA measured in rhizomes was GA_4_, as contents were 20-fold higher than the other GAs (GA_1_, GA_3_, and GA_7_). Although a trend can be observed as levels in December were lower than those of November, and rhizomes have higher contents than roots, these variations were not significant (Figure 6). Values for GA_4_ ranged from 1,800 to 2,200 ng/g DW. GA_1_ contents did not show any significant variation, although the mean in November was 92.9 ng/g DW for roots and 17 ng/g DW for rhizomes (Figure 6). GA_3_ was the GA found in the lowest concentration, since mean values for all groups were below 30 ng/g DW. Lastly, GA_7_ contents did not differ between tissues in November, but in December rhizome contents were significantly higher compared to roots (115.4 and 48.1ng/g DW, respectively). No significant difference was observed in total GA contents (GA_tot_) between roots and rhizomes. However, the ratio ABA/GAs in rhizomes was 93% higher than in roots, and this ratio further increased in December, as it increased by 68% in roots and by 48% in rhizomes.

## Discussion

Senescence represents the last phase of leaves, developmental program. It can be triggered by either internal or external factors, such as plant age or unfavorable environmental conditions. It allows plants to reallocate nutrients from photosynthetic tissues to various other organs such as younger leaves in vegetative development, or seeds during reproductive development in monocarpic plants. In perennial plants, when facing severe stress, one strategy that plants have evolved is to redistribute nutrients promoting storage in organs with meristematic tissues that remain viable and allow for regrowth once the adverse conditions are alleviated (Munné-Bosch, 2008). This is the case of *I. pseudacorus*, a perennial species that grows in wetlands and can reproduce vegetatively through its rhizomes. Leaf senescence is characterized by an organized and regulated dismantling of cellular structures and recycling of their components, with photosynthesis-related compounds being the first ones to be degraded (Matile et al., 1996). Therefore, photoinhibition and subsequent chlorophyll degradation are regarded as clear markers of leaf senescence. Coherently, in our study, maximum efficiency of PSII (*F_v_/F_m_*) was significantly lower in SL compared to NSL, and values declined in December, as temperatures decreased. Regarding total chlorophyll contents, a decrease of 45 and 40% was observed due to the senescent phenotype in November and December, respectively. When SL from December was compared to NSL from November, which was used as controls, the Chl loss accounted for a 50%. This, together with the reduction in the *F_v_/F_m_* ratio and observational studies showing that leaves from this plant died during January, indicates a clear senescence phenotype for leaves. It is important to underline the fact that this senescent phenotype in aboveground organs does not indicate the plant as an organism was senescing, but only its aboveground organs, since rhizomes establish dormancy during winter and re-establish growth during the next spring. All aboveground organs senesce in a process mediated by low temperatures combined with reduced light intensity and changes in light quality. It should be noted that the parts of leaves closest to the rhizome were the ones experiencing an accelerated senescence phenotype, which suggests that low light intensity and an increased far red-to-red ratio may interact with low temperatures in triggering leaf senescence, as it occurs in other plant species (Lim et al., 2018).

Abscisic acid and CKs are considered to be key regulators of leaf senescence. ABA has been classically considered as a promoter of leaf senescence (Samet and Sinclair, 1980; An et al., 2021b). On the other hand, studies proving CKs involvement on inhibiting chlorophyll degradation in excised leaves date back to the 1950s (Richmond and Lang, 1957). Furthermore, treatment with a mixture of CKs (zeatin and its riboside along with others) through the xylem inhibits the degradation of photosynthetic proteins and pigments in oat and wheat seedlings (Badenoch-Jones et al., 1996). Additionally, receptors such as AHK3 and CK response factors have been identified as key regulators promoting leaf longevity in response to endogenous CK accumulation in the model plant *Arabidopsis thaliana* (Kim et al., 2006; Zwack et al., 2013). Here, we observed a significant increase in the ABA/CKs ratio in senescent leaves of *I. pseudacorus* under natural conditions, most notably during December, when mean monthly maximum and minimum air temperatures decreased around 4°C relative to values recorded 1 month earlier. ABA contents increased by 72% between leaf phenotypes in November and by 75% in December. On the other hand, consistent with the literature, total cytokinin contents were lower in December than in November. Furthermore, contents of the active CK and 2-iP were non-detectable in December. Interestingly, a recent study in another species shows that exogenous application of 2-iP is effective in delaying leaf senescence (Hallmark and Rashotte, 2020), which further supports the idea that in our study leaf senescence induced by low temperatures in winter was not only modulated by increased ABA contents, but also by reduced ABA/CKs ratios, 2-iP specifically playing a role among CKs, an aspect that deserves further studies at the molecular level.

Abscisic acid is synthesized from carotenoids by the NCED enzyme. In our study, a significant decrease in the xanthophyll, violaxanthin (Vx) was observed in senescent leaves, while neoxanthin contents did not vary, suggesting that Vx was, among these two xanthophylls in leaves, the one preferentially used for ABA synthesis. Furthermore, despite rhizome accumulating ABA, the results from HPLC carotenoid quantification in subterranean organs showed non-detectable contents of ABA precursors, which suggests a carotenoid mobilization from aboveground organs (such as leaves) towards the rhizome. A study by Manzi et al. (2016) showed the inability of detached citrus roots to accumulate ABA in response to water stress, while roots of intact plants were able to do so, suggesting that ABA accumulation in subterranean organs is dependent on aerial supply. Rhizomes showed ABA contents of 0.77 and 1.15 nmol/g DW in November and December, respectively. Given that there is no re-greening of senescing organs and all aboveground organs in the next season are new sprouts and that the rhizome is responsible for leaf regrowth in spring, ABA accumulation could be providing cold tolerance in rhizomes during winter in *I. pseudacorus*, since it is known that this phytohormone induces the activation of the *cold-regulated* (COR) genes in *A. thaliana* in one of the most well-known cold-signaling pathways (Xiong et al., 2001).

Abscisic acid has been shown to play a key role in the regulation of dormancy of seeds, buds, bulbs, and tubers, in some cases interacting with gibberellins (Michalczuk, 2005; Pan et al., 2021), but no studies have focused thus far on the study of rhizome dormancy in wetland plants growing in their natural habitat. Furthermore, ABA is known to confer cold stress tolerance (Huang et al., 2017; An et al., 2021a), while gibberellins may even play a negative regulatory role, as shown by using gibberellin deficient mutants, which showed greater low temperature stress tolerance, as constitutive expression of cold-induced transcriptional activator *CBF1/DREB1b* conferred freezing tolerance as well as promoting DELLA protein accumulation in transgenic *A. thaliana* plants (Achard et al., 2008). In our study, ABA increased in roots, while gibberellin contents did not vary between root and rhizome nor between months. The only difference for gibberellins was observed in December, when rhizomes showed significantly higher GA_7_ contents than roots. In any case, the ABA-to-gibberellin ratio was always significantly higher in rhizome than roots, indicating that increasing ABA contents may be the key to overcoming low temperatures during winter. Indeed, the ratio ABA/GAs was 93% higher in rhizomes than in roots, and it further increased in December, as this ratio increased by 68% in roots and by 48% in rhizomes. These results indicate that a steep increase in ABA coupled with constant GA levels might be involved in the ability of rhizomes to sustain potential damage stemming from low temperatures and to stay viable in order to initiate the re-growth process of leaves when conditions ameliorate during the next spring.

Altogether, results from our study suggest an interplay between leaf senescence, plant developmental program, and the stress response in yellow flag plants growing in a natural wetland, in which the hormonal response below and aboveground is finely regulated and both biochemically and physiologically interconnected at the whole-plant level. It appears that ABA plays a major role both above- and belowground, not only in the regulation of leaf senescence but also in the establishment of cold tolerance in rhizomes, two processes that appear to be intimately interconnected. As rhizomes accumulated large amounts of ABA during winter, while roots did not, and neither roots nor rhizomes accumulated 9-*cis*-epoxycarotenoids, it is possible that ABA accumulation in belowground organs results from phloem transport from senescing leaves, an aspect that requires further investigation.

## Data Availability Statement

The original contributions presented in the study are included in the article/supplementary material; further inquiries can be directed to the corresponding author.

## Author Contributions

VC, AC, and SM-B conceived the experiment. VC and AC performed the experiment and biochemical analyses. VC performed the statistical analyses and wrote the manuscript with the help of SM-B. All authors contributed to the article and approved the submitted version.

## Funding

This research was supported by the Spanish Government through the PID2019-110335GB-I00 grant.

## Conflict of Interest

The authors declare that the research was conducted in the absence of any commercial or financial relationships that could be construed as a potential conflict of interest.

## Publisher’s Note

All claims expressed in this article are solely those of the authors and do not necessarily represent those of their affiliated organizations, or those of the publisher, the editors and the reviewers. Any product that may be evaluated in this article, or claim that may be made by its manufacturer, is not guaranteed or endorsed by the publisher.
